# The effects of interleukin-6-receptor inhibition on monocytes in STEMI: a substudy of the ASSAIL-MI trial

**DOI:** 10.1016/j.ebiom.2025.105960

**Published:** 2025-10-11

**Authors:** Camilla Huse, Sarah Louise Murphy, Kuan Yang, Nora Reka Balzer, Mathis K. Stokke, Anne Kristine Anstensrud, Vigdis Bjerkeli, Thiago Rentz, Prabhash Kumar Jha, Hege Katrin Ugland, Annika E. Michelsen, Thor Ueland, Sverre Holm, Ingvild Maria Tøllefsen, Bjørn Bendz, Ola Kleveland, Geir Øystein Andersen, Lars Gullestad, William E. Louch, Sindre Woxholt, Liv Osnes, Kaspar Broch, Thomas Ulas, Pål Aukrust, Peter Libby, Bente Halvorsen, Tuva B. Dahl

**Affiliations:** a Research Institute of Internal Medicine, Oslo University Hospital Rikshospitalet, Oslo, Norway; b Faculty of Medicine, Institute of Clinical Medicine, University of Oslo, Oslo, Norway; c Department of Medicine, Harvard Medical School, Boston, MA, USA; d Division of Cardiovascular Medicine, Department of Medicine, Brigham and Women's Hospital, Boston, MA, USA; e Division of Advanced Medical Science, Graduate School of Science, Technology and Innovation, Kobe University, Kobe, Japan; f Genomics and Immunoregulation, Life & Medical Sciences (LIMES) Institute, University of Bonn, Bonn, Germany; g PRECISE Platform for Single Cell Genomics and Epigenomics, German Center for Neurodegenerative Diseases and the University of Bonn, Bonn, Germany; h Institute for Experimental Medical Research, Oslo University Hospital and University of Oslo; i Department of Cardiology, Oslo University Hospital Rikshospitalet, Oslo, Norway; j Thrombosis Research Center (TREC), Division of Internal Medicine, University Hospital of North Norway, Tromsø, Norway; k Department of Cardiology, Oslo University Hospital Ullevål, Oslo, Norway; l Clinic of Cardiology, St. Olav's Hospital, Trondheim University Hospital, Trondheim, Norway; m Department of Circulation and Medical Imaging, Norwegian University of Science and Technology (NTNU), Trondheim, Norway; n Department of Immunology, Oslo University Hospital Rikshospitalet, Oslo, Norway

**Keywords:** Myocardial infarction, Interleukin 6, Monocytes, Interleukin inhibition, Suppressor of cytokine signalling 3, Chemotaxis, Apoptosis

## Abstract

**Background:**

Interleukin-6 receptor (IL-6R) inhibition by tocilizumab improves myocardial salvage index (MSI) in ST-elevation myocardial infarction (STEMI). However, the mechanisms for this effect remain unclear.

**Methods:**

This pre-defined exploratory sub-study of the ASSAIL-MI trial enumerated circulating monocytes and examined their transcriptome profile in relation to the MSI and peak troponin T (TnT) in STEMI patients randomiseded to tocilizumab (n = 101) or placebo (n = 98). RNA sequencing was performed on peripheral monocytes in 14 patients. To elaborate the *in vivo* findings, *in vitro* chemotaxis and apoptosis assays were performed on THP-1 monocytes and cardiomyocyte (HL-1) cell lines, respectively.

**Findings:**

STEMI patients had increased monocyte counts at 24 h and 3–7 days after hospitalisation/PCI and this increase was attenuated by tocilizumab. Lower monocyte levels at 24 h were associated with lower TnT levels and higher MSI. Monocyte gene expression suggested that tocilizumab modulated cytokine signalling pathways related to myocardial remodelling, apoptosis, and chemotaxis, potentially through a decrease in suppressor of cytokine signalling 3 (SOCS3). *In vitro*, tocilizumab limited apoptosis of cardiomyocytes exposed to ischemia/reperfusion and reduced chemotaxis in monocytes exposed to IL-6.

**Interpretation:**

These findings suggest that IL-6R inhibition by tocilizumab during STEMI is associated with reduced monocyte counts and cardioprotective alterations in monocyte signalling potentially linked to the downregulation of SOCS3.

**Funding:**

This work was supported by the 10.13039/501100006095South-Eastern Norway Regional Health Authority (no. 2019067) and The Research Council of Norway (no. 282867) The ASSAIL-MI main study was supported by an independent grant from ROCHE who also provided drugs/placebo for infusion.

**Research in context**

**Evidence before this study**

We have previously shown that interleukin-6 receptor (IL-6R) inhibition by a single dose of 280 mg tocilizumab prior to PCI was associated with improved myocardial salvage in patients with STEMI. More recently, we showed that these beneficial effects relate to suppressive effects on neutrophil counts and degranulation, including formation of neutrophil extracellular traps. Some studies have shown that high monocyte counts is associated with adverse outcome in STEMI patients. However, the effect of IL-6R on numbers of monocytes and the monocytes transcriptome profile in STEMI patients are at present not known.

**Added value of this study**

In the present study, we extend previously knowledge in several ways. (i) We show STEMI patients had increased monocyte counts at 24 h and 3–7 days after hospitalisation/percutaneous coronary intervention and that this increase was attenuated by tocilizumab. (ii) Lower monocyte levels at 24 h were associated with lower troponin T levels and higher myocardial salvage index. (iii) Monocyte transcriptome profiling suggested that tocilizumab modulated cytokine signalling pathways related to myocardial remodelling, apoptosis, and chemotaxis, potentially reflecting a protecting monocyte subtype that not fitted into the classical subdividing of monocyte subtypes. (iv) Our findings suggest that this tocilizumab-induced change in monocyte transcriptome profile is caused by a decrease in suppressor of cytokine signalling 3 (SOCS3). (v). IL-6R expression at hospitalisation was associated inversely with myocardial salvage index, providing further support for the benefit of reducing the IL-6 signalling pathway in STEMI. (vi) *In vitro*, tocilizumab limited apoptosis of cardiomyocytes exposed to ischemia/reperfusion and reduced chemotaxis in monocytes exposed to IL-6.

**Implications of all the available evidence**

Our findings provide evidence that tocilizumab affects monocyte counts and in particular the transcriptome profile of these cells, especially pathways related to chemotaxis and apoptosis. The study should encourage further and larger studies in of IL-6 inhibition in STEMI patients as well as in other studies where abnormal apoptosis and chemotaxis play a pathogenic role.

## Introduction

Atherosclerosis, the main cause of acute coronary syndrome (ACS), involves a bidirectional interaction between lipids and inflammation.^1^ In patients with myocardial infarction (MI), local and systemic inflammation involves a wide range of inflammatory mediators that influence clinical outcomes.^2^ While inflammation contributes to plaque destabilisation and myocardial injury through ischemia/reperfusion (I/R) damage, it also plays a critical role in infarct healing, highlighting the need for a fine balance between pro-inflammatory and anti-inflammatory, reparative, and pro-resolving responses.^2^^,^^3^

Many inflammatory cytokines rise in acute and chronic coronary artery disease (CAD).^3^^,^^4^ Interleukin (IL)-1 has drawn particular attention. The landmark “Canakinumab Antiinflammatory Thrombosis Outcome Study” (CANTOS) showed that canakinumab, a monoclonal antibody against IL-1β, reduced the risk of cardiovascular events in patients with previous MI.^5^ IL-6 also increases during MI and affects both plaque destabilisation and myocardial remodelling. In this regard, the CANTOS trial showed a particularly strong effect of canakinumab in those who achieved a reduction in circulating IL-6 concentrations.^5^

We have previously shown that tocilizumab, a humanised monoclonal antibody that blocks the IL-6 receptor (IL-6R), reduces troponin T (TnT) levels in non-ST segment elevation MI (NSTEMI).^6^ The randomiseed trial “Assessing the effect of anti-IL-6 treatment in MI” (ASSAIL-MI) showed that a single dose of tocilizumab improved the myocardial salvage index (MSI) in patients with STEMI.^7^ However, the mechanisms for the beneficial effects of tocilizumab in this setting remain incompletely understood.

Recently, we demonstrated that parts of the beneficial effects of tocilizumab in STEMI were related to a reduction of neutrophil counts and a dampening of their inflammatory potential, including attenuating effects on the formation of neutrophil extracellular traps (NETs).^8^^,^^9^ Monocytes are another cell population in the innate immune system, strongly implicated in atherogenesis and MI.^10^ Moreover, the effects of monocytes on inflammation seem at least partly to differ between the different monocyte subtypes, namely classical, intermediate and non-classical subtype. While the classical subset seems to be the most inflammatory, the non-classical subtype seems to possess a reparative potential, although this strict sub-dividing has been questioned.^11^

Suppressor of Cytokine Signalling 3 (SOCS3) plays a role in regulating immune responses in monocytes and can be induced by IL-6 as part of a regulatory feedback mechanism.^12^ However, data on how IL-6 targeted therapy modulate SOCS3 expression in monocytes are scarce and for patients with STEMI, data are lacking. It is tempting to hypothesise that tocilizumab could modulate monocyte function in STEMI as leat partly though regulation of SOCS3 expression.

This predefined sub-analysis of the ASSAIL-MI trial investigated the effects of tocilizumab on monocyte counts, transcriptomic profiles, and functional responses, with the aim to elucidate the mechanistic link between IL-6R signalling, monocyte pathology, and myocardial outcomes in STEMI.

## Methods

### Ethics

All participants initially provided oral informed consent, followed by written informed consent. The trial protocol was approved by the regional ethics committee (REK SouthEast 2016/1223). The Norwegian Medicines Agency approved the trial, which was conducted according to the Helsinki Declaration, in compliance with the principles of good clinical practice.

### Patients and study design

The ASSAIL-MI trial (Clinicaltrials.gov: NCT03004703) was a randomised clinical trial designed to test the hypothesis that a single dose of intravenous tocilizumab (280 mg) would be superior to placebo to improve myocardial salvage in patients admitted with acute STEMI. The details of the trial have been described previously.^7^^,^^13^ The primary endpoint was the MSI defined as [(area at risk - infarct size)/area at risk] x 100. The area at risk and the infarct size were assessed by magnetic resonance imaging (MRI) 3–7 days after the intervention. The trial was conducted at three high-volume PCI centres in Norway (Oslo University Hospital Rikshospitalet, Oslo University Hospital Ullevål, and St. Olav's University Hospital, Trondheim).

From March 2017 until February 2020, we enrolled 200 patients. One patient withdrew consent, leaving 199 patients for analysis. The participants were randomised to a single dose of 280 mg tocilizumab or placebo in a 1:1 ratio. The infusion began before, or simultaneously with PCI. Key inclusion criteria were STEMI and symptom onset less than 6 h before PCI. Patients with previous MI; chronic infection, or chronic autoimmune or inflammatory disease; uncontrolled inflammatory bowel disease; ongoing infectious or immunologic disease; major surgery within the past eight weeks; or treatment with immunosuppressants other than low-dose steroids (equivalent to 5 mg prednisolone per day) were excluded. Table 1 provides baseline characteristics of the study population. For the RNA sequencing analyses we also included seven healthy controls based on disease history and no use of regular medication.

**Table 1:** Baseline characteristics for the STEMI population before treatment and study drug administration.

	Tocilizumab (n = 101)	Placebo (n = 98)
**Demographics**		
Age, years	62 ± 10	60 ± 9
Men	80 (79)	87 (89)
Body mass index, kg/m^2^	27.1 ± 4.5	27.5 ± 4.3
Caucasian	99 (98)	94 (96)
**Smoking status**		
Never smokers	38 (38)	36 (37)
Previous smokers	33 (33)	24 (24)
Current smokers	30 (30)	38 (39)
**Prior conditions**		
Other vascular disease	6 (6)	6 (6)
*Aortic disease*	0	2
*Angina pectoris*	1	1
*Cerebrovascular disease*	4	2
*Peripheral vascular disease*	1	1
Diabetes mellitus	8 (8)	6 (6)
Hypertension	33 (33)	30 (31)
**Treatment**		
ACE inhibitor or ARB	22 (22)	25 (26)
Aldosterone antagonist	1 (1)	0 (0)
Oral anticoagulants	5 (5)	2 (2)
Platelet inhibitor	12 (12)	5 (5)
Beta-blocker	8 (8)	3 (3)
Calcium antagonist	13 (13)	10 (10)
Diuretic	8 (8)	8 (8)
Statin	19 (19)	9 (9)
Up-front DAPT	101 (100)	98 (100)
Time from symptom onset to arrival at PCI centre, min	151 ± 71	149 ± 72
Door-to-balloon time, min	23 ± 10	23 ± 11
**Laboratory values**		
Haemoglobin, g/l	143 ± 13	144 ± 12
Platelet count, 10^9^/l	253 ± 59	260 ± 62
Total white blood cell count, 10^9^/l	11.6 ± 3.4	11.6 ± 3.4
Aspartate transaminase, U/l	28 (22–37)	30 (24–37)
Troponin T, ng/l	44 (22–163)	49 (28–95)
CK-MB, μg/l	5.0 (2.6–14.0)	5.3 (3.0–10.0)
NT-proBNP, ng/l	79 (50–178)	63 (50–146)
Creatinine, mmol/l	74 ± 17	78 ± 20
Glucose, mmol/l	9 ± 3	9 ± 3
HbA1c, mmol/mol	37 (34–41)	37 (34–40)
Total cholesterol, mmol/l	5.3 ± 1.2	5.2 ± 1.0
HDL cholesterol, mmol/l	1.2 (0.9–1.3)	1.1 (0.9–1.3)
LDL cholesterol, mmol/l	3.7 ± 1.1	3.7 ± 0.9
C-reactive protein, mg/l	2.4 (0.9–5.0)	2.9 (1.4–5.0)
Albumin, g/l	42 ± 3	42 ± 3

Values are mean ± SD, n (%), or median (interquartile range). Note, all laboratory values including total white blood cell counts and CRP reflect values before the administration of tocilizumab.

ACE, angiotensin-converting enzyme; ARB, angiotensin receptor blocker; CK-MB, creatine kinase myocardial band; DAPT, dual anti-platelet therapy; HDL, high-density lipoprotein; LDL, low-density lipoprotein; PCI, percutaneous coronary intervention; NT-proBNP, N-terminal pro-B-type natriuretic peptide.

### Blood sampling protocol and biochemistry

Patients with STEMI arriving at the hospital for acute coronary angiography received dual antiplatelet therapy and intravenous unfractionated heparin (5000–7500 IE) before PCI. Before arrival at the hospital, most of the patients (76%) had also received unfractionated heparin (5000 IE) in the ambulance. Arterial blood samples were collected immediately before PCI, intra-arterial unfractionated heparin, and intravenous study medication were administrated at the catheterisation laboratory. Additional venous blood samples were collected at 14–33 h (24 h), at 3–7 days, at 3 months, and 6 months. Leucocyte differential counts were analysed per routine on Sysmex XN-10 (Sysmex, Kobe, Japan). High-sensitivity TnT and CRP were measured by electrochemiluminescence immunoassay (Elecsys 2010 analyser) and a MODULAR platform, respectively, both from Roche Diagnostics, Basel Switzerland. At Oslo University Hospital Rikshospitalet, we drew additional blood for the isolation of peripheral monocytes in 28 randomly selected trial participants and for an extended flow cytometry analysis in 69 randomly selected trial participants (Supplemental Table S1). In contrast to plasma sampling, monocyte isolation and storage is a time-consuming procedure, and in a setting of STEMI, we needed additional personnel for this procedure. Such personnel were only available at Oslo University Hospital. When such skilled personnel were available, we selected patients for monocyte isolation before randomisation. In addition to personnel availability, we used no other criteria for selection of patients to monocyte isolation. However, to ensure that the placebo group and the tocilizumab groups were matched in relation to demographic variables (i.e., age, BMI, hbA1c, smoking habits and blood pressure) we selected 14 patients (7 in the placebo and 7 in the tocilizumab group) out of a total of 21 where monocytes were isolated for further RNA isolation. In general, the percentage of women in the ASSAIL-MI trial was low (21% in the placebo group and 11 %in the tocilizumab group, see Table 1), and we were therefore not able to obtain a meaningful gender matching in the monocyte transcriptome analyses. Accordingly, these analyses were only performed in men.

### Isolation of monocytes

Peripheral blood mononuclear cells (PBMCs) were obtained from sodium-heparin-anticoagulated blood by Isopaque-Ficoll (Lymphoprep; Axis-Shield, Oslo, Norway) gradient centrifugation at hospitalisation (pre-PCI), 24 h (post-PCI), and after 3–7 days. PBMCs were resuspended in autoMACS® rinsing buffer (Miltenyi Biotec, Bergisch Gladbach, Germany) for positive selection through magnetic bead isolation of CD14+ cells with CD14 MicroBeads, human (Miltenyi Biotec), following the manufacturer's instructions using an autoMACS® Pro Separator (Miltenyi Biotec). Pelleted monocytes were stored at −80 °C before RNA isolation.

### RNA isolation, RNA sequencing and rt-qPCR

RNA sequencing was performed on RNA from isolated monocytes from randomly selected STEMI patients treated with placebo (n = 7) and tocilizumab (n = 7), as well as healthy controls (n = 7). Briefly, we isolated total RNA under RNAse-free conditions using Allprep DNA/RNA/Protein Mini Kit (Qiagen, Hilden, Germany) according to the manufacturer's instruction. We added 100 μM EHNA to the RLT lysis buffer. RNA concentration and purity based on the 260/230 and 260/280 ratios were determined by spectrophotometer absorbance (NanoDrop ND-1000, Thermo Fisher Scientific, Waltham, MA). We sent 0.8–1 μg total RNA to Novogene for RNA sequencing. The raw reads were trimmed with fastp (v0.20.1) in paired-end mode to remove adaptors, polyA/G tails and low-quality reads with phred score below 30.^1^ Salmon (1.4.0) was used to map the filtered reads to the human transcriptome (GENCODE H37) with 200 bootstrap iterations for transcripts quantification.^2^^,^^3^ The quantification output files were imported into DESeq2 R package by Tximeta R package. We summarised transcript-level counts to gene-level expression and compared gene expression across the treatment arms.^4^^,^^5^

Differently expressed genes (DEGs), i.e., genes that differed in expression levels between the treatment arms at an adjusted p-value <0.05, were annotated to Reactome biological pathways^6^ and the gene ontology (GO) knowledgebase^7^ using the Metascape tool.^8^ For pathway regulation analyses, raw counts of all genes were uploaded to the Reactome pathway knowledge base and analysed by the PADOG method.^9^

For rt-qPCR RNA isolated from HL-1 cell line, reverse transcription was performed using qScript cDNA supermix (Quantabio, Beverly, USA) with 600 ng RNA input for all samples, and diluted 1:10 in Nuclease-free water before qPCR. Quantitative real-time PCR was performed with Perfecta SYBR Green Supermix (Quantabio) and the CFX384 Real-time, C1000 Touch PCR system (Bio-Rad, Hercules, USA). The primers used (IL6R: F-TCCTGTGGTAGTCCATTCTCTG, R-GCCACCGTTACCCTGATTTG, gp130 F- ACCAGATTCCTGTGGACGAC, R-AGAATCCACATGCACAACCA and GAPDH: F- AACTTTGGCATTGTGGAAGG R-ACACATTGGGGGTAGGAACA were manufactured by Sigma-Merck and all used at a final concentration of 375 nM. PCR conditions were 95 °C for 3 min, followed by 40 cycles of: 95 °C for 15 s and 60 °C for 45 s. The results were analysed on the BioRad CFX Maestro Software, and qPCR Expression levels were normalised to GAPDH reference gene.

### Horizontal integration and analysis of transcriptomics datasets (hCoCena)

To investigate the impact of STEMI on monocyte gene co-expression and co-functionality, we applied the horizontal co-expression network analysis (hCoCena) analysis, as described by Oestrich et al. (2022),^10^ to RNA sequencing data from the placebo group during the acute phase (hospitalisation, 24 h post-PCI, and 3–7 days) and healthy controls. Prior to this application, we addressed the variability from unknown factors, by applying batch correction to the variance-stabilised, protein-coding gene expression data using the SVA package to estimate surrogate variables (SVs). Subsequently, we used the limma removeBatchEffect function to correct the counts based on the identified SVs. Initially, this process was performed on placebo samples collected during hospitalisation and follow-up (Supplemental Figure S1A). This corrected dataset provided the basis for hCoCena. When data from the tocilizumab group were incorporated, the batch correction was repeated, resulting in nine surrogate variables (SV1-SV9) (Supplemental Figure S2A).

The hCoCena analysis focused on the 4165 most variable genes, using a correlation coefficient cutoff of 0.714. Louvain clustering was then applied to identify gene modules within the network, which achieved a highly robust R^2^ fit of 0.924 and comprised 8186 nodes and 54,798 edges (Supplemental Figure S1B). Gene modules were defined with a minimum size threshold of 100 genes, revealing 16 distinct modules with expression patterns that significantly differed between healthy controls and placebo-treated patients in the first week post-STEMI.

To interpret the effects of tocilizumab on transcriptional changes in the monocyte population, the pre-constructed network was projected onto the complete gene expression dataset, including the tocilizumab group, using a function provided by hCoCena (Supplemental Figure S2B). Pearson correlations between numerical metadata and gene module expression were calculated to identify potential associations between clinical variables and gene expression changes. Significance was determined by an adjusted p-value (padj) < 0.05 using the Benjamini-Hochberg method.

Gene set enrichment analysis for modules such as Steelblue, Seagreen, Slategrey, Gold, Maroon, and Plum against Gene Ontology gene sets with a padj <0.05 revealed functional implications of these modules. Details of the methodology are available in Oestrich et al. (2022).^10^

### Flow cytometry

We performed an extended flow cytometry analysis of monocyte subpopulations (classical, intermediate, and non-classical) in 69 patients enrolled at Oslo University Hospital Rikshospitalet (37 allocated to tocilizumab and 32 patients allocated to placebo). Baseline characteristics of this subgroup are presented in Supplemental Table S1.

The analyses were performed at the Department of Immunology, Oslo University Hospital Rikshospitalet. The laboratory is International Standard Organisation (ISO) certified. The monocyte analysis was performed in unwashed blood samples. Briefly, EDTA-blood was incubated with optimally titrated antibodies (CD16-FITC, clone 5D2, (LSBio), CD64-PE, clone 10.2 (BioLegend), CD3-APC clone UCHT1/CD19-APC clone HIB19/CD20-APC clone 2H7/CD56-APC clone 5.1H11 (BioLegend), CD14APC-H7, clone MϕP9, (Becton Dockinson, BD), HLA-DR clone L243, (Biolegend), CD45-PO, clone H130, (Life)) for 15 min at room temperature, followed by erythrocyte lysis (BD FACSLysing Solution, Beckman Dickinson, CA). Data acquisition was performed on a Canto II Flowcytometer (BD), and FCS files analysed using Kaluza Software (Beckman Coulter). For monocyte analysis, 1 × 10^5^ cells were acquired. Monocytes were separated in classical (CD14++, CD16−), intermediate/pro-inflammatory (CD14++, CD16+) and non-classical (CD14+, CD16++).

### Western blot

Protein was isolated using Allprep DNA/RNA/Protein Mini Kit (Qiagen, Hilden, Germany) according to the manufacturer's instruction. For Western blot analysis, 20 μg of protein were separated using polyvinylacryl gel electrophoresis (SurePAGE, Genscript Biotech, Leiden, Netherlands) and transferred to a polyvinylidene difluoride membrane (PVDF, Thermo Scientific, Waltham, MA). The membranes were blocked for 1 h at room temperature using 5% non-fat dry milk and incubated overnight at 4 °C with the appropriate primary antibody. The primary antibodies used were as follows: Rabbit polyclonal to SOCS3 (1 μg/ml; detects SOCS3 at predicted molecular weight of 30 kDa) (AB16030, Abcam, Cambridge, UK), and mouse monoclonal anti-β-Actin (1:5000, detects β-Actin at predicted molecular weight of 42 kDa) (A5441, Sigma Aldrich Merck, Darmstadt, Germany). The membranes were washed 3 × 5 min each with tris-buffered saline containing Tween (TBS-T) and then incubated with horseradish peroxidase-conjugated secondary antibodies (Cell Signalling Technology, Danvers, MA) for 1 h at room temperature. After repeated washes, the immunoreactive proteins on the membrane were visualised with chemiluminescence reagents (Azure Radiance Plus). The intensities of the immunoreactive proteins were measured via computerised image analysis and normalised to β-actin.

### Apoptosis of cardiomyocytes

The atrial mouse cell line HL-1 (11) (cardiomyocytes) was cultured in flasks coated with 5 μg/ml fibronectin (Sigma Merck, Darmstadt, Germany) and 0.02% gelatin (Sigma Merck). The cells were grown in Claycomb medium (Sigma Merck) with 10% FBS, 1% penicillin/streptomycin (Sigma Merck), 0.1 mM Norepinephrine (Sigma Merck), and 2 mM l-Glutamine (Sigma Merck) at 37 °C and 5% CO2 in a humidified incubator. For the experiment, hypoxia (hypoxia chamber with 5% CO2, <1% O2, 37 °C) and starvation was applied for 24 h. Thereafter, normoxic and nutrient conditions were applied for another 24 h to mimic I/R damage during exposure to either 0.1 or 0.5 mg/ml tocilizumab or no treatment. Prior to apoptosis analysis, the detached cells were collected from the supernatant and the attached cells were detached by accutase and merged. A fluorometric TUNEL detection kit was used for apoptosis analysis according to the manufacturer's instructions (Roche Applied Science, Indianapolis, IN). Pelletts for RNA analysis was snap frozen and stored at −80C until RNA isolation.

### Chemotaxis of monocytes

THP-1 monocytes were seeded at 5000 cells/well in Incucyte® Clearview 96-well Microplates for chemotaxis (Sartorius) precoated with 50 μg/ml Matrigel Matrix Basement Membrane (growth factor reduced) (Corning, Corning, NY). The cells were activated with IL-6 (20 ng/ml, R&D Systems, Minneapolis, MN) for 1 h. Then, tocilizumab (0.5 mg/ml) was added, and the cells were incubated for 1 h before addition of monocyte chemoattractant peptide [MCP]-1/CCL2 (100 pg/ml, R&D Systems) to the lower chamber. Chemotaxis was examined by the Incucyte System with images taken once every hour in a two chamber system.

### Statistical analyses

Continuous data are presented as mean (standard deviation or standard error of the mean) or median (interquartile range) if distributions were skewed. Categorical data are reported as numbers and percentages. We used Dunnett's multiple comparisons test to investigate significant changes over time within the placebo-treated patients with STEMI. We conducted mixed-effect analysis with Bonferroni's multiple comparison test as a posthoc test to assess the effect of treatment at each time point. Correlations were calculated using Spearman's correlation coefficient. In the *in vitro* experiments, area under curve was calculated and compared by Ordinary one-way ANOVA with Tukey's multiple comparisons test. Statistical analyses were performed in GraphPad Prism 8.3.0 (GraphPad Software) or SPSS version 25 (IBM Corp., Armonk, New York). For RNA sequencing we performed FDR adjustment and reported adjusted p-values. Patient numbers (given as n) might vary over time for both counts and in the RNA analyses due to missing samples or quality issues. Missing data was evenly distributed between the treatment groups, and the missing values were assumed to be missing at random.

### Role of funders

Funders had no role in study design, data collection, data analyses, interpretation, or writing of report

## Results

### Monocyte counts increased after PCI in the placebo, but not in the tocilizumab group: data from the whole ASSAIL-MI cohort

**Fig. 1:**
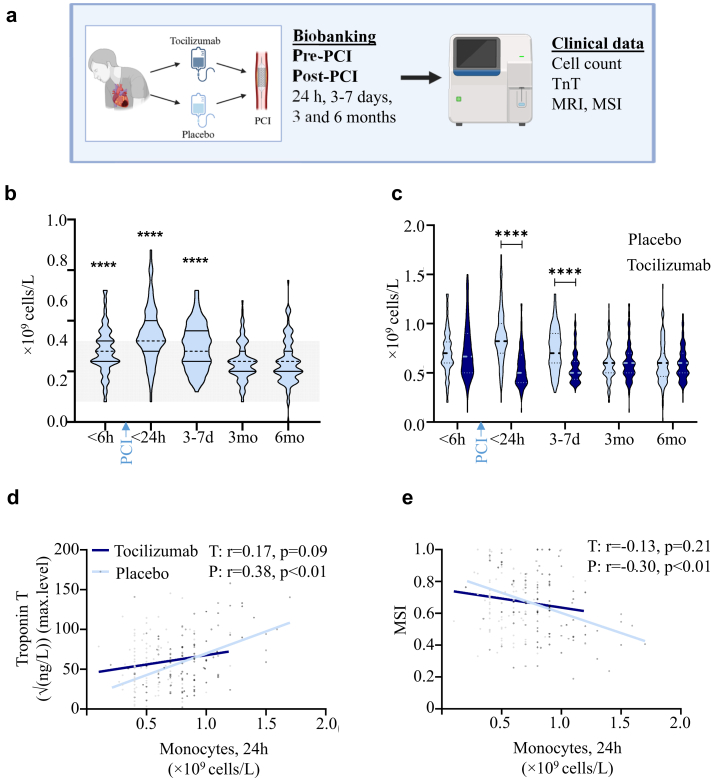
**Monocyte levels increase after PCI, but tocilizumab attenuates this effect. a**, The ASSAIL-MI study design. **b**, Monocyte levels in patients with STEMI (n = 98, only placebo) over time with SD. ∗∗∗∗p < 0.0001 compared to 3 and 6 months (mixed-effect analyses with Dunnett's multiple comparison test). Grey area is the clinical reference value for normal range. **c**, Monocyte levels in the tocilizumab (n = 101) and placebo (n = 98) group at different time points before (at hospital admission) and after PCI in patients with STEMI. ∗∗∗∗p < 0.0001 (mixed-effects analysis with a Bonferroni's multiple comparison test). **d** and **e**, The correlation between absolute monocyte counts at 24 h and TnT levels (**d**) and MSI (**e**) in tocilizumab (n = 101) and placebo (n = 80) treated patients. A trend line is indicated for easier visualisation. Correlations were calculated using Spearman's correlation coefficient (r).

In the ASSAIL-MI trial, outlined in Fig. 1a, we found that the placebo group (n = 98), had increased monocyte counts during hospitalisation with particularly high counts at 24 h after admission/PCI (Fig. 1b). In contrast, patients treated with tocilizumab before PCI (n = 101) had no increase in monocyte counts at 24 h, maintaining stable levels throughout the trial period (Fig. 1c). Classical and intermediate monocyte distribution did not differ significantly over time. There was a modestly lower level of non-classical monocytes at 24 h, but tocilizumab did not seem to influence the level of these cells (Supplemental Fig. S3A and B), suggesting that effects tocilizumab on monocytes in STEMI is not restricted to any of these monocyte subtypes.

### High monocyte counts 24 h after hospital admission were associated with decreased MSI and increased TnT in the placebo group: data from the whole ASSAIL-MI cohort

The absolute monocyte counts at 24 h after hospitalisation positively correlated with maximum TnT levels (r = 0.38, p < 0.01) and inversely with MSI (r = −0.30, p < 0.01) in the placebo group, but not in the tocilizumab group (TnT: r = 0.17, p = 0.09; MSI: r = −0.13, p = 0.21) (Fig. 1d and e). This finding might indicate a potential beneficial effect of a lower monocyte count during the acute phase of STEMI as seen in the tocilizumab group.

### Tocilizumab augmented the expression of numerous gene transcripts 24 h after hospital admission: data from randomly selected patients in the ASSAIL-MI cohort

**Fig. 2:**
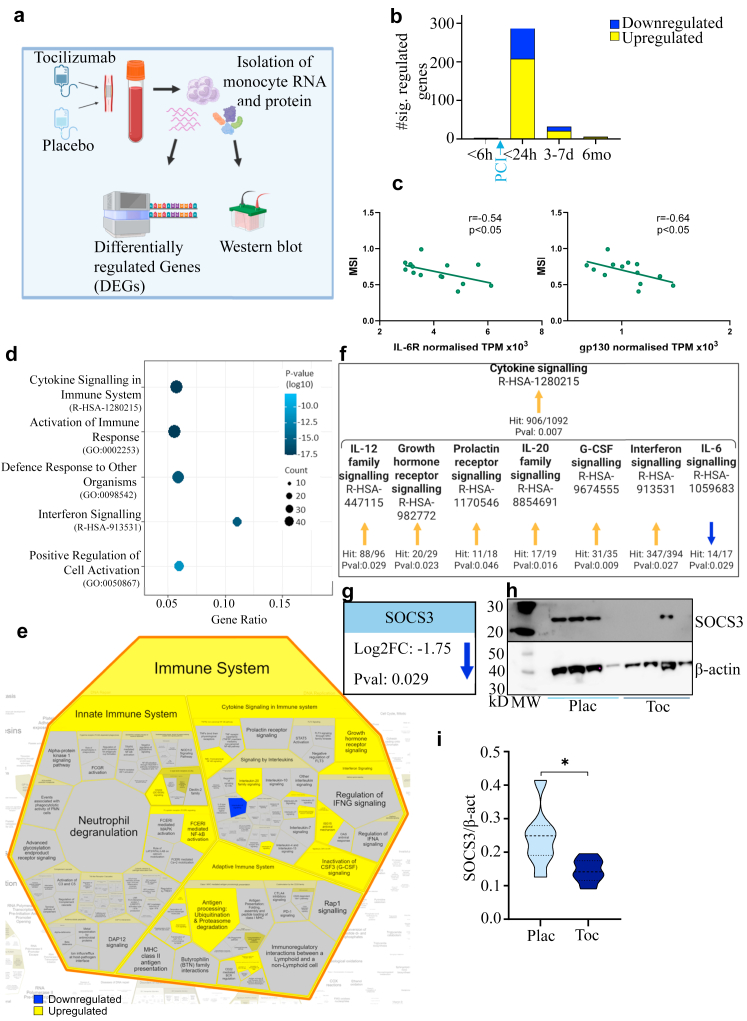
**The effect of tocilizumab on cytokine signaling in monocytes is mediated through down-regulation of suppressor of cytokine signaling 3 (SOCS3). a**, The experimental schematic for studying the effect of tocilizumab on isolated monocytes at 24 h after PCI. **b**, The number of differentially regulated genes in monocytes (adjusted p-value <0.05) between tocilizumab (n = 7) treated and placebo (n = 7) treated patients at all time points. **c**, Correlation analysis between normailized transcript level of IL-6R, gp130 at the time of hospitalisation and MSI. A trend line is indicated for easier visualisation. Correlations were calculated using Spearman's correlation coefficient (r). **d**, The top five differentially regulated pathways between monocytes isolated from STEMI patient treated with tocilizumab or placebo, 24 h after hospitalisation. Only Reactome (R-HSA) and gene ontology (GO) terms are included in the Metascape analysis. **e**, The direction of regulation of Reactome pathways under the “*Immune system*” pathway between monocytes from tocilizumab (n = 7) and placebo (n = 7) treated patients 24 h after hospitalisation. Dark blue is significantly downregulated, bright yellow is significantly upregulated in the tocilizumab arm compared to the placebo arm. The analysis is performed with the Reactome analysis tool. **f**, The upregulated Reactome pathway “Cytokine signaling” with regulated relevant sub-pathways. Yellow arrow shows significant upregulation, blue arrow shows significant downregulation. Hit rate and p-value from the analysis are given. **g**, The log2 fold change (Log2FC) and p-value for SOCS3 when comparing tocilizumab-treated patients with placebo-treated patients in the RNA sequencing analysis. Blue arrow shows significant downregulation. **h**, Representative Western blot of SOCS3 (24 kDa) and β-actin (42 kDa) for tocilizumab-treated patients (n = 4) and placebo-treated patients (n = 4). **i**, The relative protein expression of SOCS3 compared to β-actin based on the western blot for tocilizumab-treated patients (n = 7) and placebo-treated patients (n = 7). ∗p < 0.05 (unpaired t test).

To elucidate the molecular mechanisms by which tocilizumab mediated its effects on monocytes, we analysed RNA levels using RNA sequencing in isolated monocytes from a subgroup of patients (placebo group, n = 7; tocilizumab group, n = 7) Fig. 2A. Analysis of the regulation of transcripts at the different time points separately, showed only minor differences in the gene expression between the two treatment groups at hospitalisation, before treatment, with only three differently regulated genes (Fig. 2b). We hypothesised that the IL-6 receptor complex (i.e., IL-6R and gp130), the main target for tocilizumab, will be related to the primary outcome for the study, i.e., MSI. To test this hypothesis, we investigated how the RNA transcript of IL-6R and gp130 in monocytes at hospital admission correlated with MSI. Panel 2C shows that both IL-6R (r = −0.54, p = 0.04) and gp130 (r = −0.64, p = 0.01), were inversely correlated with MSI, further supporting IL-6R as a treatment target for monocytes in STEMI. In contrast to levels at hospital admission, 24 h after hospitalisation, 316 genes were differentially regulated between the two treatment groups (Fig. 2b). Of these, 208 genes were upregulated and 108 genes were downregulated in the tocilizumab group compared with the placebo group. Thereafter, the number of genes that were differentially regulated decreased to 32 (21 upregulated and 11 downregulated in the tocilizumab group) after 3–7 days and to only six (one upregulated and five downregulated in the tocilizumab group) after six months (Fig. 2b). There was little overlap between the genes that were differentially regulated at the different time points. The highest level of overlap (11 genes) was found between the measurement at 24 h and the measurement at 3–7 days after hospitalisation (Supplemental Figure S4A). The shared regulated genes are detailed in Supplemental Figure S4B.

### Pathways involved in cytokine signaling were differentially regulated in monocytes from patients treated with tocilizumab: Data from reandomly selected patients in the ASSAIL-MI cohort

Examining the differentially regulated genes 24 h after hospitalisation, a gene annotation analysis with Metascape revealed between-group differences in the regulation of several pathways involved in immune system response and inflammation (Fig. 2d). The Reactome pathway “*Cytokine Signaling in Immune System*” was the regulated term with the lowest p-value.

Further analysis using Reactome revealed a significant augmentation of the “Cytokine Signaling in the Immune System” pathway in monocytes from tocilizumab-treated patients compared to those receiving placebo, 24 h after hospitalisation (Fig. 2e). Within this overarching pathway, several sub-pathways rose significantly in the tocilizumab group during the same timeframe (Fig. 2f). These included “IL-12 family signaling,” “Growth hormone receptor signaling,” “IL-20 family signaling,” “G-CSF signaling,” and “Interferon signaling.” As expected, the “Interleukin-6 signaling” pathway, the direct target of tocilizumab, fell significantly in the tocilizumab group (Fig. 2e, blue pathway; Fig. 2f).

### SOCS3, a regulator of inflammation–RNA and protein levels fall in tocilizumab-treated patients: data from reandomly selected patients in the ASSAIL-MI cohort

SOCS3, which is induced by IL-6, inhibits all of the affected cytokine signaling pathways mentioned above,^14^ and SOCS3 rises during MI.^15^ Based on the reactome analysis in Fig. 2f and that SOCS3 pathway is a negative feedback mechanism when activating the IL-6 signaling pathway, we hypothesised that lower SOCS3 levels in the tocilizumab group could be a potential explanation for our observations in Fig. 2f. Indeed, RNA sequencing showed that SOCS3 itself had a lower expression in the tocilizumab arm (between-group difference in the fold change [log2] of −1.75 [p = 0.029]) 24 h after hospital admission (Fig. 2g). The SOCS3 protein levels of the same monocytes used for RNA-sequencing were also lower in the tocilizumab arm than in the placebo arm (Fig. 2h and i).

### Horizontal integration and analysis of transcriptomics datasets (hCoCena) in monocytes show differences between the two treatment groups: Data from reandomly selected patients in the ASSAIL-MI cohort

**Fig. 3:**
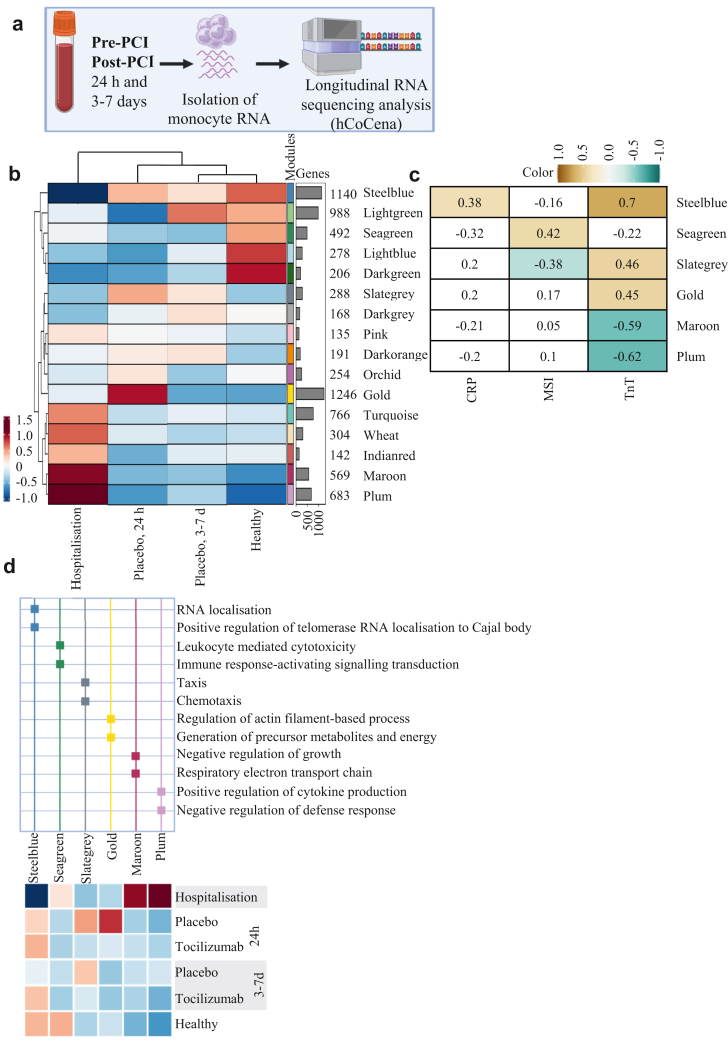
**Longitudinal gene expression analysis (hCoCena) in monocytes from patients with STEMI. a**, The experimental schematic for longitudinal RNA sequencing analysis. **b**, Heatmap over how gene transcripts from RNA sequencing from monocytes are divided into modules based on their expression pattern. This heatmap shows the module expression in the acute phase of STEMI (placebo only, n = 7) and healthy controls (n = 7). **c**, Correlation analyses between the modules and the clinical parameters CRP, MSI and TnT. We only present the modules with a significant correlation (p < 0.05). **d**, An annotation analysis of the modules from B (GO pathway) (upper half), and the expression pattern in both placebo (n = 7) and tocilizumab (n = 7) for each module (lower half). Red reflects high and blue reflects low expression.

Finally, to investigate the imprinting effects of STEMI on gene co-expression and functionality, over time we performed hCoCena analysis on RNA sequencing data from the first week following hospitalisation in the placebo group (hospitalisation, 24 h and 3–7 days post-PCI, n = 7) and healthy controls (n = 7) (Fig. 3a). This analysis identified 16 distinct gene modules with distinct and shared expression patterns between healthy controls and placebo-treated patients during the acute phase and the first-week post-STEMI (Fig. 3b). Correlating these modules with clinical outcomes such as MSI, maximum TnT, and maximum CRP, six modules (“steelblue,” “seagreen,” “slategray,” “gold,” “maroon,” and “plum”) showed significant associations (Fig. 3c).

When analysing the expression patterns in the placebo (n = 7) and tocilizumab (n = 7) groups, separately, we found that the “slategray” module was consistently downregulated by tocilizumab compared with placebo at both 24 h and 3–7 days. Similarly, the “gold” module exhibited marked downregulation by tocilizumab at 24 h (Fig. 3d, lower half). Gene ontology enrichment analysis provided insights into the biological functions of these modules and interestingly, SOCS3 was one of the genes within the “slategray” module. This module was associated with high maximum TnT and low MSI and was related to the GO term chemotaxis and importantly, this module was down-regulated by tocilizumab (Fig. 3c and d).

### Tocilizumab reduces monocyte chemotaxis: In vitro experiment

**Fig. 4:**
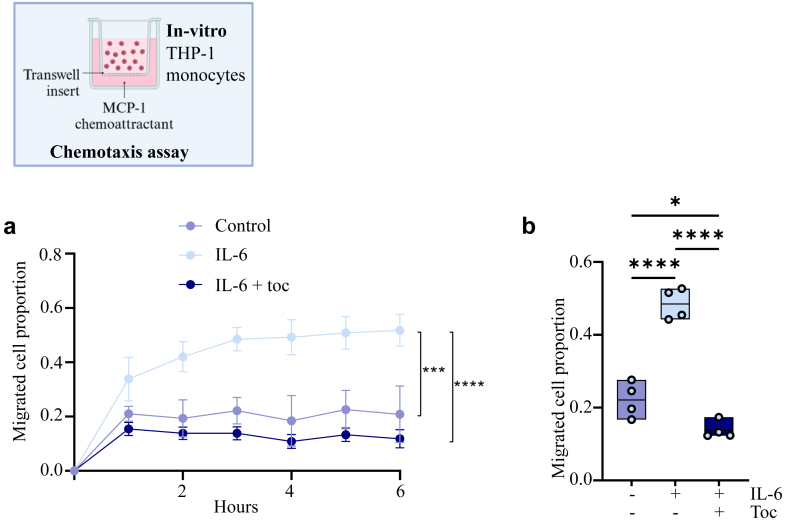
**Tocilizumab inhibits chemotaxis in monocytes.** Chemotaxis analysis with THP-1 monocytes treated with or without 20 ng/ml IL-6 and 0.5 mg/ml tocilizumab performed on the Incucyte system. **a**, The proportional area of the cells that have migrated from the top chamber. This proportion was calculated by “1-total phase object normalised to initial top value”. 100 pg/ml MCP-1 used as chemoattractant in lower chamber. The THP-1 monocytes were preincubated with IL-6 for 1 h prior to tocilizumab addition. Cells were left for 1 h before the chemoattractant was added to the lower chamber. Area under curve was calculated and compared by Ordinary one-way ANOVA with Tukey's multiple comparisons test. ∗∗∗<0.001, ∗∗∗∗<0.0001. **b**, The migrated cell propotion at 3 h. Ordinary one-way ANOVA with Tukey's multiple comparisons test. ∗<0.05, ∗∗∗∗<0.0001. Toc: tocilizumab.

Our findings in the hCoCena analysis suggest that tocilizumab modulates chemotaxis 24 h after hospitalisation, and the actual module (“slategray”) was positively associated with maximum TnT and negatively associated with MSI (Fig. 3c and d). We therefore finally tested the effect of tocilizumab on chemotaxis *in vitro*. IL-6-activated THP-1 monocytes were incubated with or without tocilizumab for 1 h before the chemoattractant (MCP-1/CCL2) was added to the lower chamber, and chemotaxis was examined by Incucyte system. Tocilizumab markedly attenuated the flux of cells towards the chamber with MCP-1, suggesting impairment of the monocytes’ chemotactic properties (Fig. 4a and b).

### Tocilizumab attenuates apoptosis in cardiomyocytes exposed to ischemia/reperfusion: In vitro experiment in a mouse cell line

**Fig. 5:**
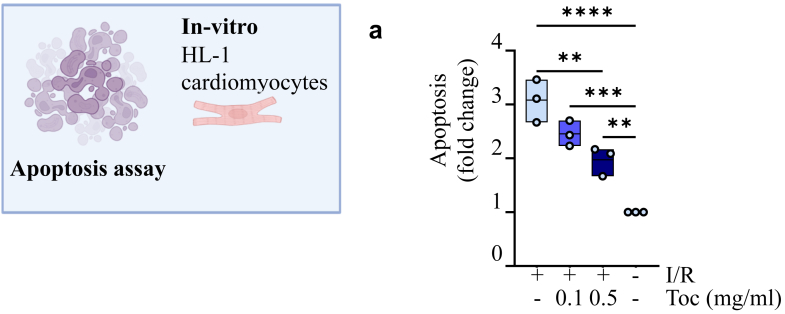
**Tocilizumab inhibits apoptosis in cardiomyocytes.** Quantitative assessment of the apoptosis at 36 h. **a**, The TUNEL assay in cardiomyocytes (atrial mouse cell line HL-1) treated with and without tocilizumab during I/R-damage. Toc: toxilizumab, CC: cholesterol crystals, I/R: ischemia/reperfusion. Ordinary one-way ANOVA with Tukey's multiple comparisons test. ∗∗<0.01, ∗∗∗<0.001, ∗∗∗∗<0.0001.

Growth hormone and members of the IL-20 family of cytokines can have anti-apoptotic effects, a feature that may improve cardiac function post–MI.^14^^,^^15^ Results from RNA sequencing showed an activation of these pathways in monocytes from patients treated with tocilizumab. We therefore next examined the ability of tocilizumab to modulate apoptosis in cells with relevance to STEMI development. We therefore examined the effects of two doses of tocilizumab on mouse cardiomyocytes. To mimic I/R, the cells were exposed to hypoxia/starvation for 24 h before normalisation of O_2_ and nutrients. As shown in Fig. 5a, tocilizumab significantly reduced the I/R-induced cardiomyocyte apoptosis in a dose-dependent manner. Gp130 is the main IL-6 receptor in cardiomyocytes. Importantly, however, IL-6R seems at least in some degree to be upregulated in cardiomyocytes during inflammation such as during I/R^16^ and notably, this was also shown in the present study when analysing IL-6R and gp130 RNA expression by rt-qPCR. Whereas gp130 in general was much stronger expressed in cardiomyocytes, we found a significant up-regulation of IL-6R RNA during ischemia/reperfusion (Supplemental Figure S5). Moreover, the gp130 receptor can interact with soluble IL-6R and induce cellular responses in cardiomyocytes through trans-signalling,^16^^,^^17^ and tocilizumab will attenuate these responses. Importantly, in the *in vitro* experiments, during reperfusion we cultured the cardiomyocytes in medium that also included 10% foetal bovine serum, which contain soluble IL-6R indicating that trans-signalling is possible in these experiments. Thus, we believe that in our *in vitro* cardiomyocyte experiment IL-6 can induce apoptosis through both classical and trans-signalling and both these effects will be attenuated by tocilizumab.

## Discussion

We have previously shown that tocilizumab reduces circulating TnT in NSTEMI,^6^ and improves myocardial salvage in STEMI.^7^ We have also showed that tocilizumab attenuate neutrophil degranulation^8^ and neutrophil extracellular trap formation,^9^ modulate N-6 methyladenosine dynamics^18^ and circular RNA profile^19^ in patients with STEMI. In this sub-study of the ASSAIL-MI trial, we present an additional possible mechanism contributing to the beneficial effects of tocilizumab, involving monocyte counts and activity. We report a markedly different gene expression pattern in monocytes 24 h after tocilizumab treatment compared with placebo, with reduced SOCS3 and increased pathways that this master regulator of cytokine signaling inhibits in the steady state. IL-6R and gp130 expression at hospitalisation is associated inversely with MSI, providing further support for the benefit of reducing the IL-6 signaling pathway in monocytes during STEMI. We demonstrated a regulation of pathways related to apoptosis and chemotaxis in monocytes from the tocilizumab-treated patients. Functional studies further supported this concept, as *in vitro*, tocilizumab attenuated I/R induced apoptosis in cardiomyocytes and reduced chemotaxis in IL-6 exposed monocytes. Our results suggest that the favourable effect of tocilizumab may derive in part from altered monocyte function during the first days following STEMI, mediated by reduced SOCS3.

STEMI and the associated I/R injury initiate an inflammatory cascade that modulates the healing of ischemically injured tissue. An excessive inflammatory reaction can also exert detrimental effects on tissue repair. Monocytes contribute to myocardial damage during MI. In this study, we showed that there was an increase in monocyte counts after PCI in the placebo group, but not in the tocilizumab group, most probably reflecting a tocilizumab-mediated impairment of inflammatory responses that again will alter monocyte trafficking. Interestingly, Meyer et al. showed a down-regulating effect of tocilizumab on monocyte counts and myocardial injury as assessed by TnT in patients with out of hospital cardiac arrest,^20^ furher support an effect of tocilumab on monocyte counts. High monocyte counts were associated with less myocardial salvage, suggesting that the lack of rise in monocyte counts in the tocilizumab group reflects beneficial effects. While several previous reports have shown increased monocyte counts during MI,^21^^,^^22^ we report the attenuating effects of tocilizumab. The effect of tocilizumab on monocytes could be secondary to attenuated inflammation, but we cannot exclude the possibility that the lower monocyte levels is due to a lesser myocardial injury and not *vice versa*, i.e., the lower monocyte count is responsible for the attenuated myocardial injury.

This study disclosed a marked difference 24 h after hospitalisation in the RNA profile in monocytes between the treatment groups. Notably, in contrast to our recent report on how most genes in neutrophils were inhibited by tocilizumab, in monocytes, most of the differentially regulated genes rose in the tocilizumab group. Several of the pathways that increased may at first glance seem harmful in MI. However, some of the signaling pathways that rose may have beneficial effects. Indeed, prolactin-, growth hormone- and G-CSF signaling may protect against ischaemic injury and adverse myocardial remodelling.^23^, ^24^, ^25^, ^26^, ^27^ Whereas members of the IL-20 family could contribute to inflammation, they also exert important roles in tissue homoeostasis.^26^ For example, studies have shown that IL-19 signaling contributes to decreased infarct size after MI,^28^ and while IL-22 can promote inflammation, it also has potent anti-apoptotic properties.^29^ Hence, whereas the signaling pathways that were up-regulated by tocilizumab may have harmful effects in STEMI, they could also exert cardioprotective actions contributing to the net beneficial effects of tocilizumab on myocardial salvage. The effect could relate to monocyte subtype, as the LIFE-Adult study showed that subtype counts were associated with the 10-year risk of cardiovascular disease. The risk was particularly enhanced for high levels of the classical monocyte subtype.^14^ Although tocilizumab did not induce a shift in these monocyte subtypes based on established flow cytometry criteria, our RNA sequencing data suggest that IL-6 inhibition induces a potential protective monocyte, hierto not classified subtype, in patients with STEMI.

In monocytes isolated from the patients, SOCS3 expression fell both at the RNA and protein levels 24 h after treatment with tocilizumab. Since IL-6 potently induces SOCS3, a master regulator of several immune pathways,^14^ this effect might have contributed to the increase in several relevant pathways (e.g. prolactin-, growth hormone-, G-CSF, and IL-20-family related pathways), which SOCS3 suppresses under normal conditions. Attenuation of SOCS3 activity may activate the IL-12 family and IFN-γ-related pathways that mediate harm during MI, but we hypothesise that the net effects of SOCS3 inhibition are beneficial in STEMI. In support of this hypothesis, SOCS3 inhibition protects against MI-induced damage in rats^30^ and I/R induced myocardial damage in mice.^31^ Moreover, cardiac-specific deletion of SOCS3 prevented adverse left ventricular remodelling following MI in mice.^32^ Moreover, SOCS3 was one of the genes within the “slategray” module that was associated with high maximum TnT and low MSI and importantly, this module was down-regulated by tocilizumab. However, regulation of SOCS3 in the setting of IL-6R inhibition during STEMI is not straightforward. IL-6 is in itself a potent inducer of SOCS3 as a counteracting mechanism to balance the IL-6 response. In fact, it is suggested that SOCS3 is the primary feedback inhibitor of IL-6 family signaling, controlling the duration of IL-6 signaling and contribute to the cellular response to IL-6.^12^ We suggest that the lower levels of SOCS3 expression in the tocilizumab as compared with the placebo arm reflect attenuated IL-6 stimulation of SOCS3 following STEMI/PCI in the placebo group, which also has been reported in cancer.^33^ However, the role of SOCS3 in the mediation of beneficial effects of tocilizumab in STEMI will require future studies that include larger clinical cohorts and regulation of these pathways within the myocardium.

This study found that tocilizumab augmented anti-apoptotic pathways and diminished chemotactic pathways at the RNA level. The tocilizumab-mediated down-regulation of the chemotactic pathways in the “slategray” module after 24 h related to a lower maximum TnT and a higher MSI. Functional studies corroborated these potential attenuating effects of tocilizumab on monocyte chemotaxis *in vitro*, and the anti-apoptotic effect was observed in I/R exposed cardiomyocytes. If these effects occur *in vivo* within the myocardium in patients with STEMI, such actions could impair cardiaomyocyte damage and infiltration of inflammatory monocytes within the myocarium, likely link to the beneficial effects of tocilizumab in patients with STEMI. Notably, tocilizumab treatment was associated with a lack of increase in monocyte counts in peripheral blood samples, which may seem incompatible with an anti-chemotactic effect on monocytes. However, inflammatory signals including inflammatory cytokines induce monocyte migration from the bone marrow and potentially also the spleen.^34^ Thus, a reduction in these signals by tocilizumab may attenuate monocyte egress from these compartments. Nonetheless, future studies should investigate whether the anti-apoptotic and anti-chemotactic effects of tocilizumab observed *in vitro* translate into beneficial effects in the myocardium *in vivo*.

Our study has several limitations. The number of patients who underwent flow cytometry of monocytes and in particular monocyte isolation for RNA sequencing was low. In general, the percentage of women in the ASSAIL-MI trial was low, and we were therefore not able to obtain a meaningful gender matching in the monocyte transcriptome analyses. Accordingly, these analyses were only performed in men, which limit the value of these data. Although the RNA sequencing results suggest that monocyte function is altered in the tocilizumab group, further functional data including *in vivo* analysis is required to support this conclusion. Moreover, we lack data on monocytes/macrophages and their functions within the myocardium, and the establishment of causal rather than correlative relationships. We also lack data on phosphorylated SOCS3 in monocytes and the expression of IL-6R and gp130 in cardiomyocytes was not shown at the protein levels.

In summary, IL-6R inhibition by tocilizumab reduces monocyte counts and induces a potential protective monocyte functions during STEMI. The IL-6R expression in monocytes upon hospital admission correlated inversely with myocardial salvage in STEMI, supporting a therapeutic role for IL-6 inhibition of monocytes during STEMI. We suggest that the beneficial effects of tocilizumab in these patients relate to altered monocyte functions that potentially could involve SOCS3 inhibition.

## Contributors

Conceptualisation; CH, AKA, TU, OK, GØA, LG, SW, KB, PA, BH, TBD, BB, PL.

Formal analysis: CH, SLM, VB, TR, PJ, HKU, AM, TU, SH, IMT, AEM, WEL, TU, TBD.

Investigation: CH, SLM, KY, NRB, MKS, AKA, VB, TR, PJ, HKU, AEM, TU, SH, IMT, WEL, LO, TU, TBD, BB, GØA, LG, SW, KB, OK.

Writing–Original Draft; CH, PA, BH, TBD.

Visualisation; CH, SLM, KY, NRB, SH, LO, TU, TBD.

Supervision: NRB, MKS, TR, PJ, TU, PA, BH, TBD, PL.

Funding acquisition; PA, BH.

All co-authors critical reviewed the manuscript and approved the submission.

## Data sharing statement

For reasons related to Norwegian legislation and the participant consent forms, the data from RNA sequencing are not available in public repositories. The data are however available upon reasonable request, following the establishment of a material and data transfer agreement between the institutions and the approval of an amendment application to the Regional Committee for Medical and Health Research Ethics to ensure that the aim of the planned research is covered by the participant consent forms.

## Declaration of interests

Kaspar Broch has received lecture fees from Pharmacosmos, AstraZeneca, Boehringer Ingelheim, Pfizer, Orion Pharma, and Vifor Pharma, and has been on advisory boards for Pfizer and AstraZeneca. Lars L. Gullestad has received lecture fees from AstraZeneca, Boehringer Ingelheim, Novartis, and Amgen. He has also been a member of the local advisory board in AstraZeneca and Boehringer Ingelheim. Annika E. Michelsen is a stock holder in Pfizer. Bente E. Halvorsen, is an SAB member in CircM, Linkøping, Sweden and is an evaluator in MH panel, Swedish Research council. Peter Libby is an unpaid consultant to, or involved in clinical trials for Amgen, Baim Institute, Beren Therapeutics, Esperion Therapeutics, Genentech, Kancera, Kowa Pharmaceuticals, Novo Nordisk, Novartis, and Sanofi-Regeneron. Peter Libby is a member of the scientific advisory board for Amgen, Caristo Diagnostics, CSL Behring, Elucid Bioimaging, Kancera, Kowa Pharmaceuticals, Olatec Therapeutics, Novartis, PlaqueTec, Polygon Therapeutics, TenSixteen Bio, Soley Therapeutics, and XBiotech, Inc. Peter Libby's laboratory has received research funding in the last 2 years from Novartis, Novo Nordisk and Genentech. Peter Libby is on the Board of Directors of XBiotech, Inc. Peter Libby has a financial interest in Xbiotech, a company developing therapeutic human antibodies, in TenSixteen Bio, a company targeting somatic mosaicism and clonal haematopoiesis of indeterminate potential (CHIP) to discover and develop novel therapeutics to treat age-related diseases, and in Soley Therapeutics, a biotechnology company that is combining artificial intelligence with molecular and cellular response detection for discovering and developing new drugs, currently focussing on cancer therapeutics. Peter Libby's interests were reviewed and are managed by Brigham and Women's Hospital and Mass General Brigham by their conflict-of-interest policies.
