# Supplementary material for: The effects of interleukin-6-receptor inhibition on monocytes in STEMI: a substudy of the ASSAIL-MI trial
Source: eBioMedicine. 2025 Oct 11;121:105960. doi: 10.1016/j.ebiom.2025.105960 (PMC12547449; doi:10.1016/j.ebiom.2025.105960)
Supplement: Supplemental Material [file mmc1.pdf]

Online Appendix

The effects of interleukin-6-receptor inhibition on monocytes in STEMI: a substudy of the ASSAIL-MI trial

Table of Contents

Supplemental Table 1: Baseline characteristics of the populations included in flow cytometry analysis before treatment 1

Supplemental Figure 1: Principal component analysis and degree distribution plot for the hCoCena analysis with placebo and healthy 2

Supplemental Figure 2: Principal component analysis, degree distribution plot, and hCoCena module heatmap for placebo and tocilizumab....3

Supplemental Figure 3: Level of classical, intermediate, and non-classical monocytes following STEMI, and effect of tocilizumab treatment....4

Supplemental Figure 4: Shared genes between different time points between monocytes from placebo and tocilizumab-treated patients....5

Supplemental Figure 5: IL-6R and gp130 are expressed in the HL1 cardiomyocyte cell line……………………...6

Supplemental Table 1: Baseline characteristics of the populations included in flow cytometry analysis before treatment

Variable	Tocilizumab (n=37)	Placebo (n=32)
Demography		
Age – years	61 ± 10	59 ± 9
Men – no (%)	30 (81)	30 (94)
Body mass index – kg/m^2	27.0 ± 4.6	27.5 ± 3.2
Caucasian race – no (%)	37 (100)	31 (97)
Smoking status – no (%)		
Never smokers	16 (43)	17 (53)
Previous smokers	13 (35)	5 (16)
Current smokers	8 (22)	10 (31)
Prior conditions – no (%)		
Angina Pectoris	0 (0)	1 (3)
Cerebrovascular disease	1 (3)	0 (0)
Diabetes mellitus	3 (8)	0 (0)
Hypertension	9 (24)	6 (19)
Previous myocardial infarction	0 (0)	0 (0)
Prior CABG	0 (0)	0 (0)
Prior PCI	0 (0)	1 (3)
Treatment – no (%)		
ACE* inhibitor or ARB[†]	6 (16)	6 (19)
Aldosterone antagonist	0 (0)	0 (0)
Oral anticoagulants	2 (5)	0 (0)
Platelet inhibitor	3 (8)	1 (3)
Beta blocker	4 (11)	0 (0)
Calcium antagonist	4 (11)	2 (6)
Diuretic	3 (8)	3 (9)
Statin	5 (14)	4 (13)
Up-front DAPT[‡]	37 (100)	32 (100)
Time from symptom onset to arrival at PCI centre – min	156 ± 78	162 ±76
Door-to-balloon time – min	20 ± 9	18 ± 5
Infarct location		
Left anterior descending branch	15 (41)	10 (31)
Circumflex or marginal	4 (11)	6 (19)
Right coronary artery	16 (43)	15 (47)
Other	2 (5)	1 (3)
Laboratory values		
Haemoglobin – g/l	144 ± 11	145 ± 8
Platelet count – 10^9/l	252 ± 57	269 ± 71
Total white blood cell count – 10^9/l	11.7 ± 3.0	11.7 ± 3.1
Aspartate transaminase – U/l	30 (21-43)	33 (25-43)
Troponin T – ng/l	45 (22-170)	52 (24-125)
CK-MB[§] – μg/l (placebo minus 1)	5.0 (2.7-16.0)	5.9 (3.2-13.3)
NT-proBNP[‖] – ng/l	67 (50-460)	62 (50-159)
Creatinine – mmol/l	71 ± 13	79 ± 16
Glucose – mmol/l	8 (7-10)	8 (7-10)
HbA1c – mmol/mol	36 (34-41)	37 (35-39)
Total cholesterol – mmol/l	5.5 ± 1.4	5.2 ± 1.0
HDL[¶] cholesterol – mmol/l	1.2 (1.1-1.4)	1.2 (0.9-1.4)
LDL[#] cholesterol – mmol/l	3.8 ± 1.0	3.8 ± 0.9
C-reactive protein – mg/l	1.9 (0.8-4.1)	2.5 (1.2-5.8)
Albumin – g/l	42 ± 3	42 ± 3

Baseline characteristics stratified by treatment allocation. Values are presented as mean ± SD, median (interquartile range) or number (%) as appropriate. *ACE = angiotensin-converting enzyme; †ARB = angiotensin receptor blocker; ‡DAPT = dual anti-platelet therapy; §CK-MB = creatine kinase myocardial band; || NT-proBNP = N-terminal pro-B-type natriuretic peptide; ¶HDL = high-density lipoprotein; #LDL = low-density lipoprotein. For continuous variables, we used a one-way ANOVA or a Kruskal-Wallis test depending on the distribution. Categorical variables were tested using Chi-square. There were no differences between the two treatment arms of STEMI patients.

A

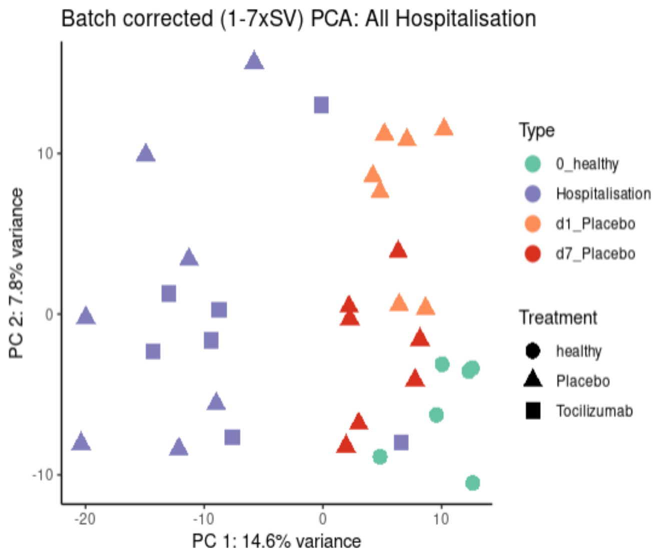

B

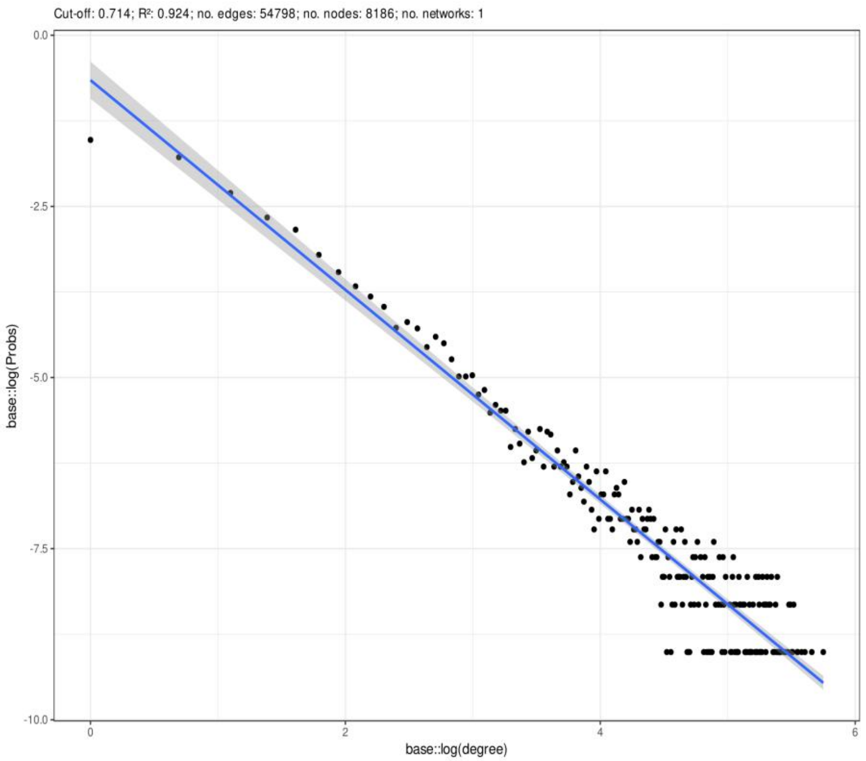

Supplemental Figure 1: Principal component analysis and degree distribution plot for the hCoCena analysis with placebo and healthy. A, Principal component analysis (PCA) plots of normalised, protein-coding monocyte gene expression counts. PCA plot after normalised counts were batch corrected with surrogate variables (SV) 1-7 out of the 10 SVs detected. **B**, Degree distribution plot, visualising the logarithmic degree distribution and linear regression after applying the given correlation cutoff on the batch corrected normalised counts. Confidence interval is displayed in grey. Healthy (n=6), placebo (n=7).

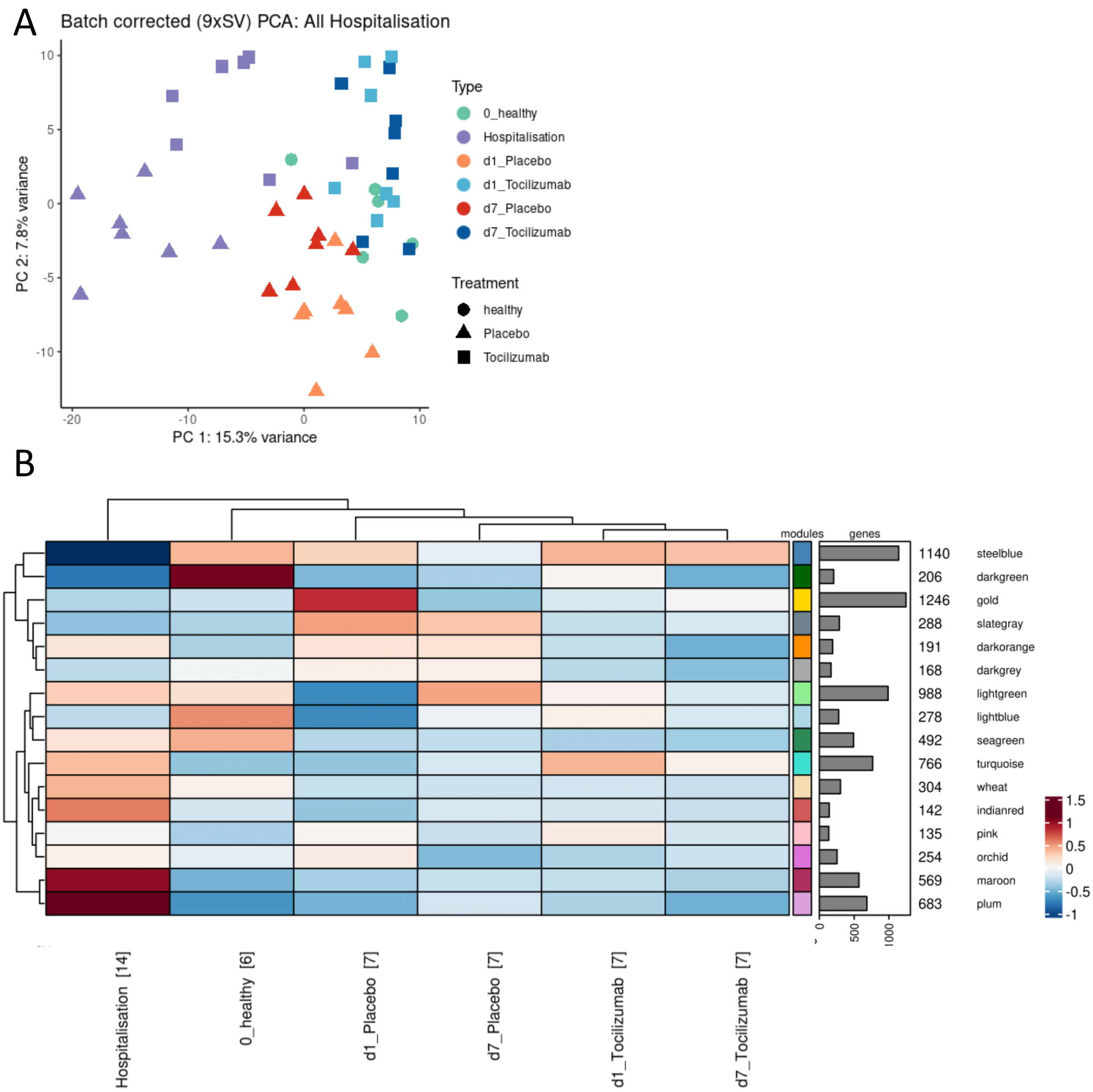

Supplemental Figure 2: **Principal component analysis, degree distribution plot, and hCoCena module heatmap for placebo and tocilizumab. A**, Principal component analysis (PCA) plots of normalised, protein-coding monocyte gene expression counts. The normalised counts were batch corrected with surrogate variables (SV) 1-9 out of the 10 SVs detected. **B**, hCoCena module heatmap representing the transcriptional changes caused by tocilizumab after the treatment arm was added to the gene network initially determined by Placebo treatment arm and healthy controls. Healthy (n=6), tocilizumab (n=7), placebo (n=7).

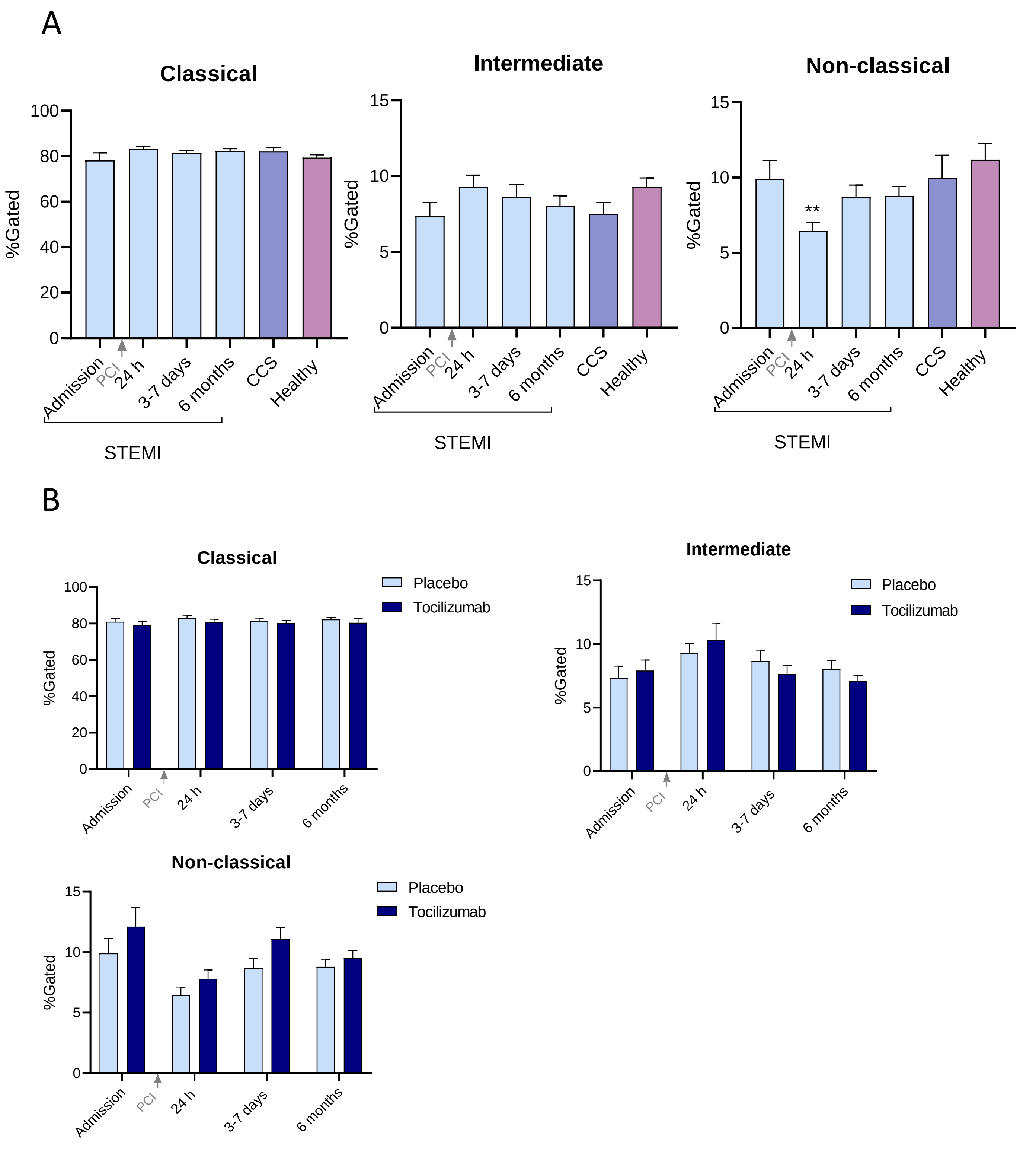

Supplemental Figure 3: Level of classical, intermediate, and non-classical monocytes following STEMI, and effect of tocilizumab treatment. A, The level of classical, intermediate, and non-classical monocytes in patients presenting with STEMI (n = 32, placebo only), compared to patients with chronic coronary syndrome (CCS) (n = 20) and healthy controls (n = 20). **p < 0.01 versus healthy (One-way ANOVA with Dunnett's multiple comparison test to investigate significant differences between SAP or healthy controls compared to the STEMI-group). Data are given as mean and SEM. Hospital admission was within 6 hours after symptom debut. **B**, The level of the monocyte subgroups in the placebo (n = 32) and the tocilizumab (n = 37) treated patients. (mixed-effects analysis with a Bonferroni's multiple comparison test).

A

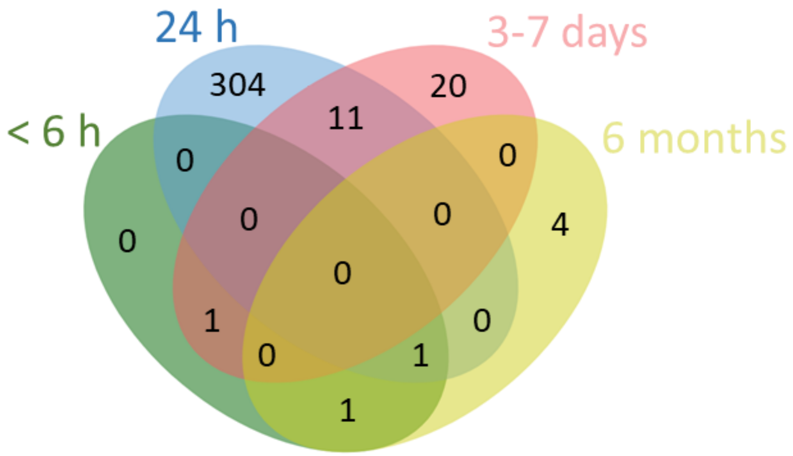

B

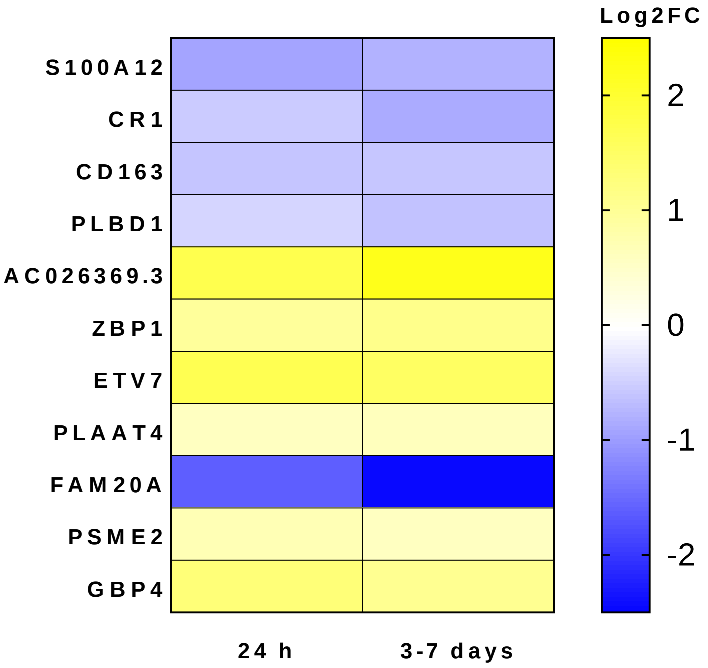

Supplemental Figure 4: Shared genes between different time points between monocytes from placebo and tocilizumab-treated patients. A, Venn diagram of shared genes that are regulated at the different time points between placebo and tocilizumab in monocytes. **B**, The 11 genes that are shared between 24 hours and 3-7 days and their log2 fold change (Log2 FC) for the two time points.

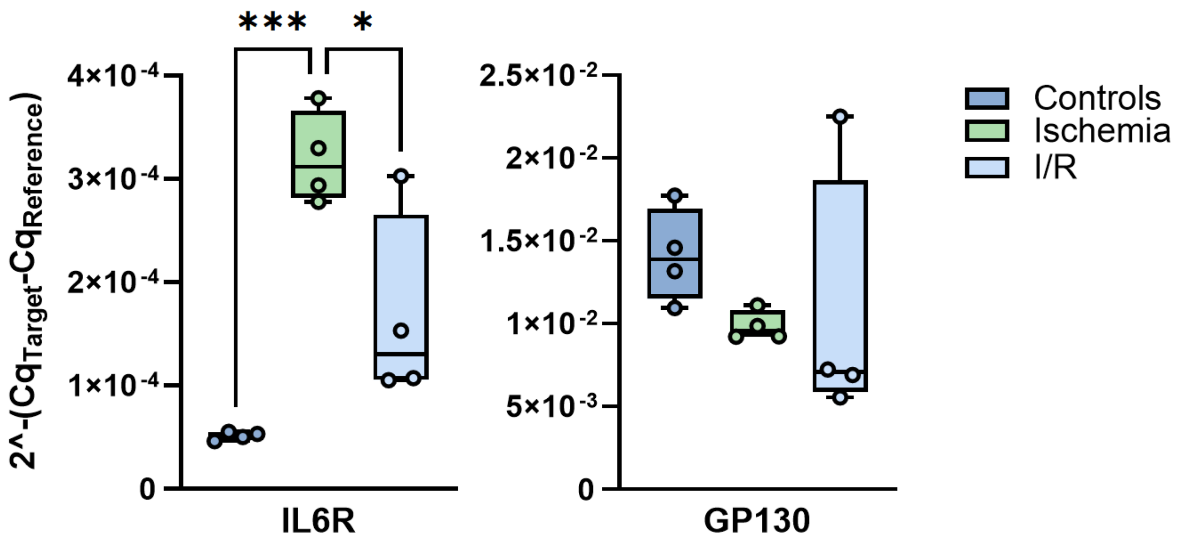

Supplemental Figure 5: IL-6R and gp130 are expressed in the HL1 cardiomyocyte cell line. Rt-qPCR of IL-6R and gp130 in the HL1 cardiomyocyte cell line in an I/R model. When related to the reference gene GAPDH, there is a markedly higher expression of the g130 co-receptor compared to IL-6R. Panels A and B show IL-6R and gp130 RNA expression in cardiomyocytes without and with ischemia and with ischemia/reperfusion (I/R). * $p<0.05$, ***$p<0.0001$. n=4.
